# Screening Performance of Digital Breast Tomosynthesis vs Digital Mammography in Community Practice by Patient Age, Screening Round, and Breast Density

**DOI:** 10.1001/jamanetworkopen.2020.11792

**Published:** 2020-07-28

**Authors:** Kathryn P. Lowry, Rebecca Yates Coley, Diana L. Miglioretti, Karla Kerlikowske, Louise M. Henderson, Tracy Onega, Brian L. Sprague, Janie M. Lee, Sally Herschorn, Anna N. A. Tosteson, Garth Rauscher, Christoph I. Lee

**Affiliations:** 1 Department of Radiology, University of Washington, Seattle Cancer Care Alliance, Seattle; 2 Kaiser Permanente Washington Health Research Institute, Seattle; 3 Division of Biostatistics, Department of Public Health Sciences, University of California Davis, Davis; 4 Department of Medicine, University of California, San Francisco; 5 Department of Epidemiology and Biostatistics, University of California, San Francisco; 6 Department of Radiology, University of North Carolina, Chapel Hill; 7 Department of Biomedical Data Science, Geisel School of Medicine at Dartmouth, Lebanon, New Hampshire; 8 Department of Surgery, University of Vermont Cancer Center, University of Vermont Larner College of Medicine, Burlington; 9 Department of Radiology, University of Vermont Cancer Center, University of Vermont Larner College of Medicine, Burlington; 10 The Dartmouth Institute for Health Policy and Clinical Practice and Norris Cotton Cancer Center, Geisel School of Medicine at Dartmouth, Lebanon, New Hamsphire; 11 Division of Epidemiology and Biostatistics, School of Public Health, University of Illinois at Chicago, Chicago

## Abstract

**Importance:**

Digital mammography (DM) and digital breast tomosynthesis (DBT) are used for routine breast cancer screening. There is minimal evidence on performance outcomes by age, screening round, and breast density in community practice.

**Objective:**

To compare DM vs DBT performance by age, baseline vs subsequent screening round, and breast density category.

**Design, Setting, and Participants:**

This comparative effectiveness study assessed 1 584 079 screening examinations of women aged 40 to 79 years without prior history of breast cancer, mastectomy, or breast augmentation undergoing screening mammography at 46 participating Breast Cancer Surveillance Consortium facilities from January 2010 to April 2018.

**Exposures:**

Age, Breast Imaging Reporting and Data System breast density category, screening round, and modality.

**Main Outcomes and Measures:**

Absolute rates and relative risks (RRs) of screening recall and cancer detection.

**Results:**

Of 1 273 492 DM and 310 587 DBT examinations analyzed, 1 028 891 examinations (65.0%) were of white non-Hispanic women; 399 952 women (25.2%) were younger than 50 years; and 671 136 women (42.4%) had heterogeneously dense or extremely dense breasts. Adjusted differences in DM vs DBT performance were largest on baseline examinations: for example, per 1000 baseline examinations in women ages 50 to 59, recall rates decreased from 241 examinations for DM to 204 examinations for DBT (RR, 0.84; 95% CI, 0.73-0.98), and cancer detection rates increased from 5.9 with DM to 8.8 with DBT (RR, 1.50; 95% CI, 1.10-2.08). On subsequent examinations, women aged 40 to 79 years with heterogeneously dense breasts had improved recall rates and improved cancer detection with DBT. For example, per 1000 examinations in women aged 50 to 59 years, the number of recall examinations decreased from 102 with DM to 93 with DBT (RR, 0.91; 95% CI, 0.84-0.98), and cancer detection increased from 3.7 with DM to 5.3 with DBT (RR, 1.42; 95% CI, 1.23-1.64). Women aged 50 to 79 years with scattered fibroglandular density also had improved recall and cancer detection rates with DBT. Women aged 40 to 49 years with scattered fibroglandular density and women aged 50 to 79 years with almost entirely fatty breasts benefited from improved recall rates without change in cancer detection rates. No improvements in recall or cancer detection rates were observed in women with extremely dense breasts on subsequent examinations for any age group.

**Conclusions and Relevance:**

This study found that improvements in recall and cancer detection rates with DBT were greatest on baseline mammograms. On subsequent screening mammograms, the benefits of DBT varied by age and breast density. Women with extremely dense breasts did not benefit from improved recall or cancer detection with DBT on subsequent screening rounds.

## Key Points

**Question:**

Does screening performance of digital breast tomosynthesis vs digital mammography differ by breast density, age, or screening round in community practice?

**Findings:**

In this comparative effectiveness study assessing 1 584 079 screening examinations across 46 US facilities, the largest improvements in recall and cancer detection rates with digital breast tomosynthesis were observed on baseline screens. On subsequent screens, both recall and cancer detection rates improved using digital breast tomosynthesis vs digital mammography for women aged 40 to 79 years with heterogeneously dense breasts and for women aged 50 to 79 years with scattered fibroglandular density; by contrast, performance was similar in women with extremely dense breasts.

**Meaning:**

Women undergoing baseline screening may benefit most from digital breast tomosynthesis, whereas on subsequent screens, the benefits of digital breast tomosynthesis may vary by age and density category.

## Teaser

This comparative effectiveness study evaluates the screening recall and cancer detection rates of digital mammography vs digital breast tomosynthesis stratified by age, baseline screening, subsequent screening round, and breast density category in a large US cohort of women aged 40 to 79 years.

## Introduction

Digital breast tomosynthesis (DBT) has rapidly disseminated for routine breast cancer screening, with evidence of improved overall screening performance with DBT when compared with digital mammography (DM).^1,2,3,4,5,6^ Use of DBT has steadily increased since its approval by the US Food and Drug Administration in 2011,^7^ with 63% of Mammography Quality Standards Act–certified facilities reporting DBT units in 2019.^8^ Although this uptake has been associated with growing evidence that DBT improves screening recall or cancer detection,^1,3,6,9,10^ the magnitude of these improvements varies across studies and by screening setting.^5^ Many questions about the benefits of DBT remain unanswered, for example, how outcomes vary by breast density or age or vary on baseline vs subsequent rounds of screening. These questions are important to address during this period of transition to DBT while many centers are currently unable to offer DBT to all women undergoing screening. Self-reported physician surveys suggest that many clinicians preferentially perform DBT in women with dense breasts and other breast cancer risk factors;^11,12^ however, data are insufficient to support these practices. Moreover, this information is needed to support informed decision-making for women undergoing screening, many of whom pay for additional DBT screening costs out of pocket owing to inconsistent insurance coverage.^13^

Density-specific performance of DBT is particularly important to characterize in the current era of density legislation, as women are increasingly being notified of their density category and the limitations of mammography for women with dense breasts. To date, 38 states have passed legislation requiring that screening mammography reports include language notifying all women with dense breasts (defined as Breast Imaging Reporting and Data System [BI-RADS] categories *heterogeneously dense* or *extremely dense*) about these limitations.^14^ In 2019, a federal bill was passed directing the US Food and Drug Administration to develop national standards for density notification in mammography reports.^15^ However, despite the increasing use of DBT, it is not known how its screening performance may vary by density category because few studies have compared performance of DM vs DBT across BI-RADS density groups. The purpose of the present study is to evaluate differences in screening performance for DM vs DBT for women by density category, age group, and baseline vs subsequent screening round among women undergoing screening in Breast Cancer Surveillance Consortium (BCSC) facilities in community practice.

## Methods

This study was compliant with the Health Insurance Portability and Accountability Act. Registries and the Statistical Coordinating Center received a federal Certificate of Confidentiality and other protections for the identities of women, physicians, and facilities. This study followed the International Society for Pharmacoeconomics and Outcomes Research (ISPOR) reporting guideline of good research practices for comparative effectiveness research: defining, reporting and interpreting nonrandomized studies of treatment effects using secondary data sources. The participating BCSC registries and Statistical Coordinating Center obtained institutional review board approval for participant enrollment, linkage and pooling of data, and data analysis via passive consent (3 registries) or waiver of written consent (2 registries and the Statistical Coordinating Center). No one received compensation or was offered any incentive for participating in this study.

### Study Setting and Data Sources

Data were prospectively collected by participating facilities across 5 regional BCSC imaging registries whose screening populations collectively are broadly representative of the general US population:^16,17^ Carolina Mammography Registry, New Hampshire Mammography Network, Vermont Breast Cancer Surveillance System, San Francisco Mammography Registry, and Metropolitan Chicago Breast Cancer Registry. The registries prospectively collect woman-level and examination-level information from a mix of academic and community facilities with linkage to pathology databases and state or regional tumor registries to allow for complete cancer capture. For the present study, facilities with fewer than 100 DM or DBT screening examinations performed during the study period were excluded because previous work from the BCSC has shown performance differences between facilities that offer DBT screening and those that do not.^18^ Facilities without information on availability of prior examinations for comparison were also excluded.

### Participants and Examinations

Our study cohort included women aged 40 to 79 years receiving screening with DM or DBT from January 2010 to April 2018 at a participating BCSC facility. Women with a history of mastectomy, personal history of breast cancer, or breast augmentation were excluded.

### Measures and Definitions

At all facilities, demographic and breast health history information were collected from women at the time of their examination via questionnaire, including personal or family history of breast cancer, menopausal status, and time since last mammogram. Information about prior breast biopsies was collected via self-report, electronic health records, and pathology databases. Five-year breast cancer risk was calculated using the BCSC risk calculator, version 2.^19^

Breast density was classified by radiologists based on mammographic assessment using the following BI-RADS density categories: almost entirely fatty, scattered fibroglandular density, heterogeneously dense, and extremely dense.^20,21^ Screening outcomes were classified based on BI-RADS assessment categories of 0 (incomplete, needs additional imaging evaluation), 1 (negative), 2 (benign), 3 (probably benign), 4 (suspicious for malignancy), and 5 (highly suspicious for malignancy).^20,21^ Rates of recall, biopsy recommendations, and total and invasive cancer detection were calculated per 1000 screening examinations performed. Screening recall rate was defined as examinations with an initial BI-RADS assessment of 0, 3, 4, or 5, and the biopsy recommendation rate was defined as the number of examinations with final assessment category of 4 or 5, per standard BI-RADS audit definitions.^20,21^ Screen-detected cancers were defined as invasive or in situ breast cancers diagnosed within 90 days of screening recall. Baseline examinations were defined as any first mammography examination (DM or DBT); subsequent examinations were defined as examinations with any prior mammography examination (DM or DBT).

### Statistical Analysis

Examination-level demographic characteristics and risk factors were summarized for first and subsequent DM and DBT examinations. Multivariable log-link regression was used to estimate relative risks (RR) of each outcome by modality for subgroups defined by breast density, age, and screening round. Regression models were estimated using generalized estimating equations^22^ with robust covariance estimates to account for nonnested clustering^23^ of examinations within woman and radiologist. Interactions between modality, screening round, breast density, and age were included to estimate variability in the modality for each combination of examination characteristics. Regression models were also adjusted for woman- and examination-level characteristics, including race/ethnicity, family history of breast cancer, prior breast biopsy, 5-year breast cancer risk, time since last mammogram, year of examination, and BCSC registry.

Absolute adjusted rates of recall, biopsy recommendation, total cancer detection, and invasive cancer detection were estimated using marginal standardization^24^ to reflect the distribution of examination-level characteristics within each subgroup defined by breast density, age, and screening round. Estimates for baseline examinations were adjusted for the distribution of breast density observed in first examinations for each age group. Standard errors were estimated using 1000 bootstrap resamples of the coefficient and covariance estimates from regression models.

Estimates are presented stratified by age and density for all subsequent examinations; estimates for baseline examinations are stratified by age only because breast density is not known prior to the baseline examination. Because screening recall rates and biopsy recommendation rates included both true-positive and false-positive examinations, we present ratios of screening recalls and biopsy recommendations to cancers detected to reflect trade-offs of screening benefits and harms. All analyses were performed using SAS, version 9.4 (SAS Institute Inc), and R, version 3.4.4 (R Foundation for Statistical Computing).

## Results

Our analysis included 1 584 079 screening examinations performed at 46 facilities (Table 1): 1 273 492 (80.4%) DM, and 310 587 (19.6%) DBT; 1 028 891 examinations (65.0%) in white non-Hispanic women; 399 952 examinations (25.2%) in women younger than 50 years; and 671 136 examinations (42.4%) in women with heterogeneously dense or extremely dense breasts. Of subsequent screening examinations, 1 327 311 women (88.9%) had a prior screening examination within 2 years. Five-year BCSC risk scores were low/average (<1.67%) in 1 003 422 examinations (63.3%), and high (≥2.5%) in 136 490 examinations (8.6%).

**Table 1.** Demographic and Clinical Characteristics of 1 584 079 Participants in the Screening Cohort

Characteristic	No. (%) of participants				
	Total (N = 1 584 079)	Baseline screen		Subsequent screen	
		DM (N = 74 307)	DBT (N = 17 095)	DM (N = 1 199 185)	DBT (N = 293 492)
Age group, y					
40-49	399 952 (25.2)	41 581 (56.0)	9744 (57.0)	279 052 (23.3)	69 575 (23.7)
50-59	525 479 (33.2)	17 375 (23.4)	3883 (22.7)	403 898 (33.7)	100 323 (34.2)
60-69	438 105 (27.7)	11 129 (15.0)	2635 (15.4)	340 269 (28.4)	84 072 (28.6)
70-79	220 543 (13.9)	4222 (5.7)	833 (4.9)	175 966 (14.7)	39 522 (13.5)
BI-RADS breast density					
Almost entirely fat	162 359 (10.2)	7599 (10.2)	1568 (9.2)	128 274 (10.7)	24 918 (8.5)
Scattered fibroglandular density	679 518 (42.9)	27 688 (37.3)	6850 (40.1)	519 910 (43.4)	125 070 (42.6)
Heterogeneously dense	557 463 (35.2)	30 906 (41.6)	7239 (42.3)	407 654 (34.0)	111 664 (38.0)
Extremely dense	113 673 (7.2)	5706 (7.7)	1280 (7.5)	84 299 (7.0)	22 388 (7.6)
Unknown	71 066 (4.5)	2408 (3.2)	158 (0.9)	59 048 (4.9)	9452 (3.2)
Race/ethnicity					
Asian	141 949 (9.0)	8120 (10.9)	1792 (10.5)	117 613 (9.8)	14 424 (4.9)
Black non-Hispanic	224 160 (14.2)	16 722 (22.5)	2165 (12.7)	186 626 (15.6)	18 647 (6.4)
Latina	98 945 (6.2)	8881 (12.0)	1610 (9.4)	76 324 (6.4)	12 130 (4.1)
Mixed/other	22 147 (1.4)	1586 (2.1)	346 (2.0)	15 943 (1.3)	4272 (1.5)
Native American	4646 (0.3)	525 (0.7)	149 (0.9)	3153 (0.3)	819 (0.3)
Pacific Islander	406 (0.0)	43 (0.1)	16 (0.1)	276 (0.0)	71 (0.0)
White non-Hispanic	1 028 891 (65.0)	32 110 (43.2)	9758 (57.1)	753 225 (62.8)	233 798 (79.7)
Unknown	62 935 (4.0)	6320 (8.5)	1259 (7.4)	46 025 (3.8)	9331 (3.2)
First degree family history of breast cancer					
Yes	259 058 (16.4)	6796 (9.1)	1653 (9.7)	198 967 (16.6)	51 642 (17.6)
No	1 271 418 (80.3)	66 207 (89.1)	14 815 (86.7)	976 706 (81.4)	213 690 (72.8)
Unknown	53 603 (3.4)	1304 (1.8)	627 (3.7)	23 512 (2.0)	28 160 (9.6)
Prior breast biopsy					
No	1 148 021 (72.5)	69 986 (94.2)	15 991 (93.5)	884 191 (73.7)	177 853 (60.6)
Yes	314 346 (19.8)	4235 (5.7)	1050 (6.1)	242 361 (20.2)	66 700 (22.7)
Unknown	121 712 (7.7)	86 (0.1)	54 (0.3)	72 633 (6.1)	48 939 (16.7)
Time since prior mammogram, y					
≤2	1 327 311 (83.8)	0	0	1 060 363 (88.4)	266 948 (91.0)
3-4	81 693 (5.2)	0	0	65 616 (5.5)	16 077 (5.5)
≥5	49 019 (3.1)	0	0	40 118 (3.3)	8901 (3.0)
No previous mammogram	91 402 (5.8)	74 307 (100)	17 095 (100)	0	0
Unknown	34 654 (2.2)	0	0	33 088 (2.8)	1566 (0.5)
BCSC 5-y risk					
<1.00%	439 400 (27.7)	44 459 (59.8)	10 494 (61.4)	318 825 (26.6)	65 622 (22.4)
1.00%-1.66%	564 022 (35.6)	19 690 (26.5)	4604 (26.9)	435 673 (36.3)	104 055 (35.5)
1.67%-2.49%	289 896 (18.3)	5220 (7.0)	1294 (7.6)	217 776 (18.2)	65 606 (22.4)
2.50%-3.99%	119 169 (7.5)	947 (1.3)	261 (1.5)	87 852 (7.3)	30 109 (10.3)
≥4.00%	17 321 (1.1)	61 (0.1)	15 (0.1)	12 300 (1.0)	4945 (1.7)
Missing	154 271 (9.7)	3930 (5.3)	427 (2.5)	126 759 (10.6)	23 155 (7.9)
Year of examination					
2010-2012	663 755 (41.9)	34 247 (46.1)	864 (5.1)	614 081 (51.2)	14 563 (5.0)
2013-2015	707 497 (44.7)	33 799 (45.5)	9126 (53.4)	510 030 (42.5)	154 542 (52.7)
2016-2018	212 827 (13.4)	6261 (8.4)	7105 (41.6)	75 074 (6.3)	124 387 (42.4)
Prior DBT examination					
No	1 435 527 (90.6)	74 307 (100)	17 095 (100)	1 176 488 (98.1)	167 637 (57.1)
Yes	148 552 (9.4)	0 (0)	0 (0)	22 697 (1.9)	125 855 (42.9)

Abbreviations: BCSC, Breast Cancer Surveillance Consortium; BI-RADS, Breast Imaging and Reporting Data System; DBT, digital breast tomosynthesis; DM, digital mammography.

Adjusted rates and relative risks of screening outcomes for DBT vs DM by breast density category, age group, and baseline vs subsequent screening round are presented in Table 2 (recall and biopsy recommendation rates) and Table 3 (total and invasive cancer detection rates), with absolute differences for recall and total cancer detection depicted in Figure, A and B (unadjusted rates are available in eTable in the Supplement). Across all ages and breast density categories, absolute benefits of DBT relative to DM were greatest for baseline examinations, with lower recall rates and higher cancer detection rates. Per 1000 baseline examinations, screening recalls decreased from 240 for DM to 215 for DBT in women aged 40 to 49 years (RR, 0.90; 95% CI, 0.80-1.01), from 241 for DM to 204 for DBT in women aged 50 to 59 years (RR, 0.84; 95% CI, 0.73-0.98), and from 219 for DM to 178 for DBT in women aged 60 to 79 years (RR, 0.80; 95% CI, 0.69-0.95) (Table 2). Total cancer detection rates per 1000 examinations increased from 3.2 for DM to 4.4 for DBT in women aged 40 to 49 years (RR, 1.41; 95% CI, 1.11-1.80), from 5.9 for DM to 8.8 for DBT in women aged 50 to 59 years (RR, 1.50; 95% CI, 1.10-2.08), and from 10.8 for DM to 15.1 for DBT in women aged 60 to 79 years (RR, 1.42; 95% CI, 1.09-1.86) (Table 3). Approximately 75% of cancers detected were invasive, with higher invasive cancer detection with DBT across age groups (eg, from 4.2 to 6.5 [RR, 1.55; 95% CI, 1.13-2.09] per 1000 examinations in women aged 50 to 59 years).

**Table 2.** Adjusted Absolute Rates per 1000 Screening Examinations and Relative Risks of Screening Recall and Biopsy Recommendation^a^

BI-RADS density	Recall rate				Biopsy recommendation rate			
	aAR (95% CI)		Difference (95% CI)	Relative Risk (95% CI)	aAR (95% CI)		Difference (95% CI)	Relative Risk (95% CI)
	DM	DBT			DM	DBT		
Baseline screening								
All women aged 40-49 y	240 (227 to 255)	215 (192 to 243)	−24.9 (−47.9 to −0.3)	0.90 (0.80 to 1.01)	35.1 (30.8 to 40.0)	46.6 (40.0 to 54.5)	11.45 (5.87 to 17.92)	1.32 (1.16 to 1.52)
All women aged 50-59 y	241 (223 to 261)	204 (177 to 238)	−36.9 (−63.7 to −5.1)	0.84 (0.73 to 0.98)	41.4 (36.4 to 47.1)	54.5 (46.3 to 64.3)	13.12 (6.44 to 21.23)	1.31 (1.15 to 1.51)
All women aged 60-79 y	219 (202 to 240)	178 (153 to 211)	−41.1 (−63.9 to −12.1)	0.80 (0.69 to 0.95)	40.5 (35.8 to 45.6)	49.7 (43.4 to 56.8)	9.21 (4.88 to 14.68)	1.23 (1.12 to 1.37)
Subsequent screening								
Aged 40-49 y								
Almost entirely fat	58 (50 to 66)	49 (40 to 60)	−9.1 (−18.2 to 0.7)	0.84 (0.70 to 1.01)	11.1 (9.6 to 13.0)	10.6 (8.7 to 13.1)	−0.41 (−2.65 to 1.87)	0.96 (0.78 to 1.17)
Scattered fibroglandular density	103 (97 to 111)	80 (73 to 89)	−22.8 (−30.4 to −15.0)	0.78 (0.71 to 0.85)	13.7 (12.5 to 15.1)	13.8 (12.2 to 15.7)	0.13 (−1.65 to 2.05)	1.01 (0.89 to 1.15)
Heterogeneously dense	132 (124 to 139)	119 (108 to 130)	−12.8 (−22.7 to −2.8)	0.90 (0.83 to 0.98)	17.1 (15.4 to 18.9)	20.6 (18.5 to 23.0)	3.52 (1.21 to 5.79)	1.21 (1.07 to 1.35)
Extremely dense	112 (103 to 120)	122 (113 to 131)	10.2 (−0.7 to 21.6)	1.09 (0.99 to 1.20)	17.9 (16.0 to 20.4)	19.7 (17.2 to 22.5)	1.78 (−1.54 to 4.96)	1.10 (0.92 to 1.29)
Aged 50-59 y								
Almost entirely fat	46 (41 to 52)	39 (33 to 46)	−7.3 (−13.5 to −0.4)	0.84 (0.72 to 0.99)	10.0 (8.6 to 11.7)	10.1 (8.5 to 12.1)	0.10 (−1.86 to 2.03)	1.01 (0.83 to 1.22)
Scattered fibroglandular density	82 (76 to 88)	69 (63 to 75)	−13.3 (−19.4 to −7.5)	0.84 (0.77 to 0.90)	13.0 (11.9 to 14.4)	13.8 (12.5 to 15.3)	0.77 (−0.56 to 2.20)	1.06 (0.96 to 1.18)
Heterogeneously dense	102 (96 to 108)	93 (85 to 101)	−9.0 (−16.5 to −1.5)	0.91 (0.84 to 0.98)	16.1 (14.6 to 17.8)	20.4 (18.4 to 22.4)	4.27 (2.33 to 6.38)	1.27 (1.14 to 1.41)
Extremely dense	91 (84 to 98)	93 (81 to 105)	1.7 (−10.1 to 13.6)	1.02 (0.89 to 1.15)	16.5 (14.6 to 18.8)	19.0 (16.0 to 22.6)	2.54 (−0.82 to 5.79)	1.15 (0.95 to 1.37)
Aged 60-79 y								
Almost entirely fat	51 (46 to 56)	39 (33 to 47)	−11.5 (−18.9 to −4.1)	0.77 (0.65 to 0.92)	10.8 (9.5 to 12.4)	10.4 (8.8 to 12.6)	−0.39 (−2.36 to 1.66)	0.96 (0.79 to 1.15)
Scattered fibroglandular density	74 (70 to 80)	61 (56 to 67)	−13.1 (−18.0 to −8.3)	0.82 (0.76 to 0.89)	13.8 (13.0 to 14.7)	13.9 (12.7 to 15.4)	0.14 (−1.08 to 1.45)	1.01 (0.92 to 1.11)
Heterogeneously dense	87 (81 to 93)	77 (71 to 85)	−9.9 (−16.6 to −3.2)	0.89 (0.81 to 0.96)	16.6 (15.5 to 17.9)	20.0 (18.5 to 22.0)	3.45 (1.80 to 5.35)	1.21 (1.10 to 1.33)
Extremely dense	64 (57 to 71)	62 (51 to 74)	−2.3 (−13.4 to 10.1)	0.96 (0.80 to 1.16)	16.9 (15.4 to 18.9)	18.7 (15.9 to 21.6)	1.71 (−1.34 to 4.85)	1.10 (0.93 to 1.30)

Abbreviations: aAR, adjusted absolute rate; BI-RADS, Breast Imaging Reporting and Data System; DBT, digital breast tomosynthesis; DM, digital mammography.

^a^ Rates and relative risks are adjusted for the woman- and examination-level characteristics given in Table 1, including race/ethnicity, family history of breast cancer, history of prior biopsy, 5-year risk of breast cancer, time since prior mammogram, and year of examination.

**Table 3.** Adjusted Absolute Rates and Relative Risks of Total Cancer Detection and Invasive Cancer Detection^a^

BI-RADS density	Total cancer detection rate				Invasive cancer detection rate			
	aAR (95% CI)		Difference (95% CI)	Relative risk (95% CI)	aAR (95% CI)		Difference (95% CI)	Relative Risk (95% CI)
	DM	DBT			DM	DBT		
Baseline screening								
All women aged 40-49 y	3.2 (2.7 to 3.9)	4.4 (3.5 to 5.7)	1.28 (0.38 to 2.39)	1.41 (1.11 to 1.80)	2.2 (1.7 to 2.7)	3.1 (2.3 to 4.1)	0.93 (0.21 to 1.83)	1.44 (1.10 to 1.90)
All women aged 50-59 y	5.9 (4.9 to 7.2)	8.8 (6.3 to 12.6)	2.89 (0.60 to 6.15)	1.50 (1.10 to 2.08)	4.2 (3.3 to 5.3)	6.5 (4.7 to 8.8)	2.28 (0.57 to 4.36)	1.55 (1.13 to 2.09)
All women aged 60-79 y	10.8 (9.3 to 12.9)	15.1 (11.7 to 20.2)	4.27 (0.75 to 8.54)	1.42 (1.09 to 1.86)	9.0 (7.4 to 11.2)	12.3 (9.8 to 16.0)	3.29 (0.41 to 6.40)	1.40 (1.07 to 1.79)
Subsequent screening								
Aged 40-49 y								
Almost entirely fat	1.6 (1.4 to 1.9)	1.6 (1.1 to 2.1)	−0.08 (−0.52 to 0.41)	0.95 (0.69 to 1.27)	1.1 (1.0 to 1.3)	1.1 (0.8 to 1.5)	−0.06 (−0.37 to 0.34)	0.95 (0.69 to 1.30)
Scattered fibroglandular density	2.2 (2.0 to 2.5)	2.4 (1.9 to 2.9)	0.19 (−0.34 to 0.78)	1.08 (0.85 to 1.37)	1.5 (1.3 to 1.7)	1.6 (1.3 to 2.1)	0.12 (−0.26 to 0.57)	1.08 (0.83 to 1.40)
Heterogeneously dense	2.5 (2.2 to 2.7)	3.1 (2.5 to 3.9)	0.68 (0.05 to 1.43)	1.28 (1.02 to 1.61)	1.6 (1.5 to 1.8)	2.1 (1.7 to 2.7)	0.51 (0.05 to 1.07)	1.31 (1.03 to 1.67)
Extremely dense	2.5 (2.2 to 2.9)	2.7 (2.0 to 3.5)	0.17 (−0.53 to 0.98)	1.07 (0.79 to 1.42)	1.4 (1.2 to 1.7)	1.5 (1.1 to 2.2)	0.09 (−0.39 to 0.80)	1.06 (0.74 to 1.60)
Aged 50-59 y								
Almost entirely fat	2.1 (1.9 to 2.5)	2.3 (1.6 to 3.0)	0.13 (−0.53 to 0.90)	1.06 (0.76 to 1.45)	1.6 (1.4 to 1.9)	1.7 (1.3 to 2.4)	0.12 (−0.34 to 0.73)	1.08 (0.80 to 1.49)
Scattered fibroglandular density	3.3 (3.1 to 3.6)	4.0 (3.4 to 4.6)	0.68 (0.08 to 1.35)	1.21 (1.02 to 1.42)	2.4 (2.2 to 2.6)	3.0 (2.5 to 3.5)	0.55 (0.04 to 1.12)	1.23 (1.02 to 1.48)
Heterogeneously dense	3.7 (3.4 to 4.0)	5.3 (4.6 to 6.0)	1.56 (0.90 to 2.31)	1.42 (1.23 to 1.64)	2.6 (2.4 to 2.9)	3.9 (3.5 to 4.6)	1.29 (0.73 to 1.97)	1.49 (1.26 to 1.76)
Extremely dense	3.7 (3.3 to 4.3)	4.4 (3.3 to 6.0)	0.71 (−0.46 to 2.28)	1.19 (0.89 to 1.61)	2.3 (2.0 to 2.7)	2.8 (2.0 to 3.8)	0.49 (−0.35 to 1.58)	1.21 (0.86 to 1.72)
Aged 60-79 y								
Almost entirely fat	3.8 (3.3 to 4.4)	3.9 (2.9 to 5.1)	0.15 (−0.85 to 1.33)	1.04 (0.79 to 1.35)	3.1 (2.7 to 3.6)	3.1 (2.3 to 4.2)	0.02 (−0.73 to 0.99)	1.01 (0.77 to 1.33)
Scattered fibroglandular density	5.5 (5.2 to 5.9)	6.5 (5.7 to 7.4)	1.00 (0.17 to 1.89)	1.18 (1.03 to 1.35)	4.3 (4.1 to 4.7)	5.0 (4.4 to 5.8)	0.64 (−0.06 to 1.41)	1.15 (0.99 to 1.33)
Heterogeneously dense	6.1 (5.7 to 6.5)	8.5 (7.6 to 9.4)	2.38 (1.54 to 3.23)	1.39 (1.25 to 1.54)	4.6 (4.3 to 5.1)	6.5 (5.8 to 7.3)	1.82 (1.01 to 2.66)	1.39 (1.21 to 1.59)
Extremely dense	5.9 (5.3 to 6.7)	6.9 (5.5 to 8.7)	0.97 (−0.54 to 2.82)	1.16 (0.91 to 1.49)	3.9 (3.4 to 4.6)	4.4 (3.4 to 6.0)	0.51 (−0.71 to 2.22)	1.13 (0.84 to 1.59)

Abbreviations: aAR, adjusted absolute rate; BI-RADS, Breast Imaging Reporting and Data System; DBT, digital breast tomosynthesis; DM, digital mammography.

^a^ Rates and relative risks are adjusted for the woman- and examination-level characteristics given in Table 1, including race/ethnicity, family history of breast cancer, history of prior biopsy, 5-year risk of breast cancer, time since prior mammogram, and year of examination.

**Figure.**
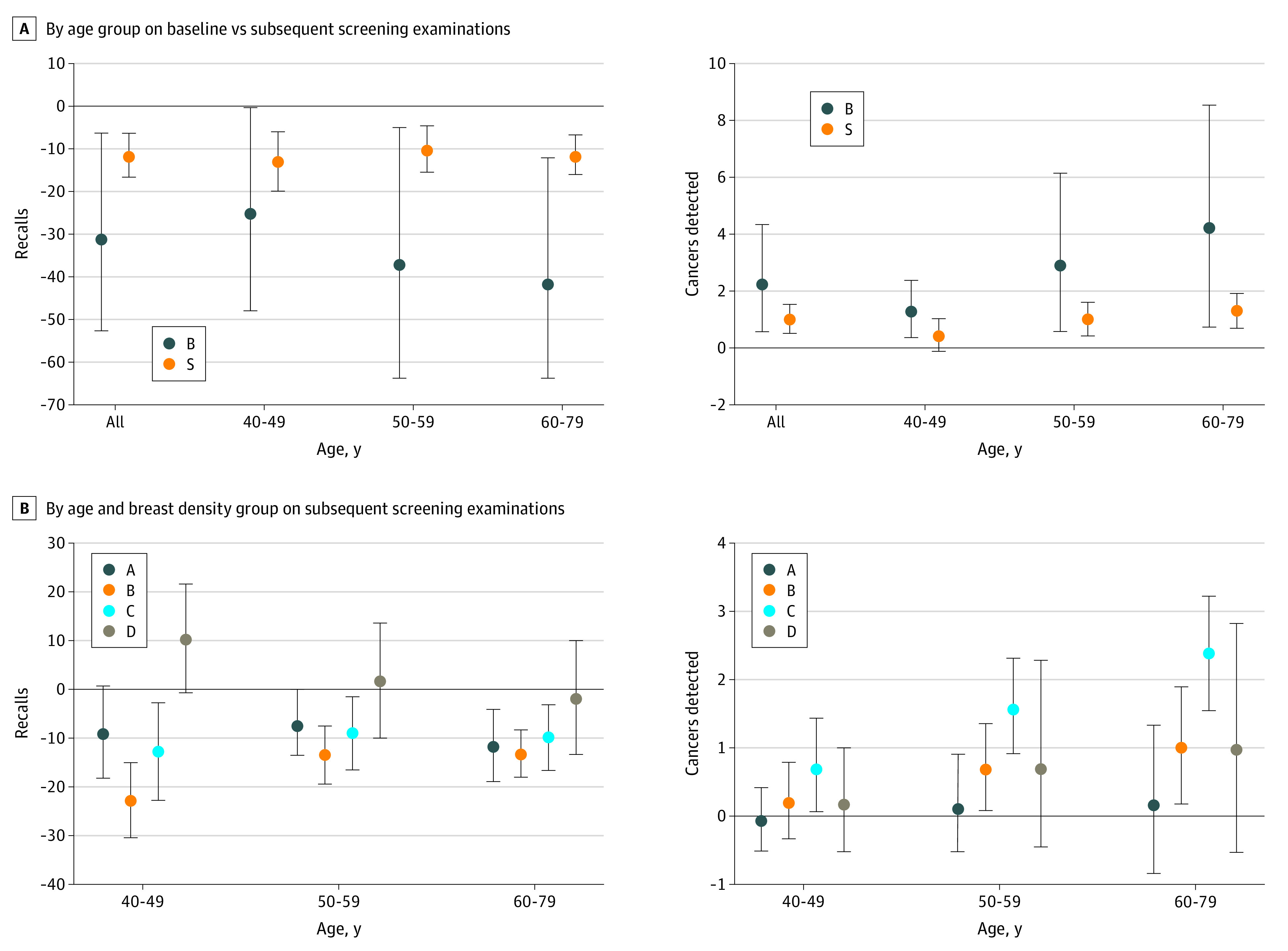
Absolute Differences in Recall and Total Cancer Detection Rates for Digital Breast Tomosynthesis vs Digital Mammography Values are expressed as absolute differences in rates for digital breast tomosynthesis relative to digital mammography per 1000 screening examinations. In panel A, B represents baseline examination; S, subsequent examination. In panel B, Breast Imaging and Reporting Data System breast density categories are A, almost entirely fat; B, scattered fibroglandular density; C, heterogeneously dense; and D, extremely dense. Error bars indicate 95% CIs.

Among subsequent screening examinations, differences in screening performance between DBT and DM varied by age and density subgroups. Recall rate was lower with DBT for women with scattered fibroglandular density and heterogeneously dense breasts in all age groups, with the largest reduction in women aged 40 to 49 years with scattered fibroglandular density (103 for DM to 80 for DBT per 1000 examinations [RR, 0.78; 95% CI, 0.71-0.85]). In women aged 50 to 59 years with scattered fibroglandular density, the number of recall examinations decreased from 102 with DM to 93 with DBT (RR, 0.91; 95% CI, 0.84-0.98). Screening recall was also lower for DBT in women aged 50 to 79 years with almost entirely fatty density classifications. By contrast, there were no significant differences in recall rates in women with extremely dense breasts in any age group.

Cancer detection rates were consistently higher for subsequent DBT examinations compared with DM in women with heterogeneously dense breasts, with absolute gains increasing with age: total cancer detection per 1000 examinations increased from 2.5 to 3.1 in women aged 40 to 49 years (RR, 1.28; 95% CI, 1.02-1.61), from 3.7 to 5.3 in women aged 50 to 59 (RR, 1.42; 95% CI, 1.23-1.64), and from 6.1 to 8.5 in women aged 60 to 79 years (RR, 1.39; 95% CI, 1.25-1.54). Similarly, invasive cancer detection was higher with DBT in women with heterogeneously dense breasts across all age groups. Total cancer detection rates were also higher for DBT than for DM in women with scattered fibroglandular density at 50 to 59 years of age (3.3 vs 4.0; RR, 1.21; 95% CI, 1.02-1.42) and 60 to 79 years of age (5.5 vs 6.5; RR, 1.18; 95% CI, 1.03-1.35); invasive cancer detection rates were higher at 50 to 59 years of age (2.4 vs 3.0; RR, 1.23; 95% CI, 1.02-1.48). Cancer detection rates were not significantly different for women with almost entirely fatty or extremely dense breasts across all age groups.

Differences in biopsy recommendation rates mirrored differences in cancer detection rates and were higher for DBT than for DM on baseline examinations in all age groups. For example, on baseline exams, biopsy recommendation rates per 1000 examinations increased for women aged 40 to 49 years from 35.1 for DM to 46.6 for DBT (RR, 1.32; 95% CI, 1.16-1.52). On subsequent examinations, biopsy recommendation rates were higher for DBT than for DM in women with heterogeneously dense breasts but were similar for other density categories.

Numbers of screening recalls per cancer detected and biopsies recommended per cancer detected decreased with increasing age group for both DM and DBT (Table 4). In general, the ratio of recalls to cancers detected was lower for DBT than for DM for baseline and subsequent examinations although these differences did not meet statistical significance in all age and density groups. These ratios were most improved on baseline examinations and on subsequent examinations for women with heterogeneously dense breasts. The ratio of biopsies recommended to cancers detected was similar for DM and DBT on both baseline and subsequent examinations, and for all age and density subgroups.

**Table 4.** Adjusted Ratios of Screening Recalls per Cancers Detected and Biopsies per Cancers Detected^a^

BI-RADS density	Adjusted ratio (95% CI)			
	Recalls per cancer detected		Biopsies per cancer detected	
	DM	DBT	DM	DBT
Baseline screening				
All women aged 40-49 y	75.0 (61.7-92.3)	48.9 (37.1-64.1)	11.0 (8.6-14.0)	10.6 (7.9-13.9)
All women aged 50-59 y	40.8 (32.8-50.3)	23.2 (16.3-32.4)	7.0 (5.3-8.9)	6.2 (4.3-8.6)
All women aged 60-79 y	20.3 (16.5-24.5)	11.8 (8.7-15.6)	3.7 (3.0-4.6)	3.3 (2.5-4.3)
Subsequent screening				
Aged 40-49 y				
Almost entirely fat	36.2 (28.9-42.7)	30.6 (23.1-43.7)	6.9 (5.5-8.4)	6.6 (4.6-10.7)
Scattered fibroglandular density	46.8 (42.1-52.7)	33.3 (26.7-42.7)	6.2 (5.5-7.1)	5.8 (4.5-7.5)
Heterogeneously dense	52.8 (47.9-60.1)	38.4 (30.1-47.5)	6.8 (6.1-8.0)	6.6 (5.0-8.6)
Extremely dense	44.8 (37.9-52.7)	45.2 (34.2-62.7)	7.2 (6.0-8.5)	7.3 (5.6-10.1)
Aged 50-59 y				
Almost entirely fat	21.9 (18.3-26.1)	17.0 (12.0-24.9)	4.8 (3.9-5.8)	4.4 (3.0-6.7)
Scattered fibroglandular density	24.8 (22.7-27.6)	17.2 (14.4-20.7)	3.9 (3.6-4.4)	3.5 (3.0-4.1)
Heterogeneously dense	27.6 (24.7-30.6)	17.5 (15.2-20.4)	4.4 (3.8-5.0)	3.8 (3.3-4.5)
Extremely dense	24.6 (21.1-27.7)	21.1 (14.6-29.6)	4.5 (3.7-5.3)	4.3 (3.0-6.1)
Aged 60-79 y				
Almost entirely fat	13.4 (11.2-16.3)	10.0 (7.3-14.0)	2.8 (2.4-3.5)	2.7 (1.8-4.0)
Scattered fibroglandular density	13.5 (12.3-14.9)	9.4 (8.0-11.0)	2.5 (2.3-2.7)	2.1 (1.9-2.5)
Heterogeneously dense	14.3 (12.9-15.8)	9.1 (8.0-10.4)	2.7 (2.5-3.0)	2.4 (2.1-2.7)
Extremely dense	10.8 (9.2-12.8)	9.0 (6.6-12.1)	2.9 (2.4-3.3)	2.7 (2.0-3.6)

Abbreviations: BI-RADS, Breast Imaging Reporting and Data System; DBT, digital breast tomosynthesis; DM, digital mammography.

^a^ Adjusted for woman- and examination-level characteristics given in Table 1, including race/ethnicity, family history of breast cancer, history of prior biopsy, 5-year risk of breast cancer, time since prior mammogram, and year of examination.

## Discussion

In this study, we evaluated screening performance of DM vs DBT across 46 facilities participating in 5 regional BCSC registries and compared the outcomes of DBT by age and breast density, and by baseline vs subsequent rounds of screening. Our results highlight important differences in screening benefits of DBT among specific groups of women and screening scenarios, with the largest benefits for both recall reduction and improved cancer detection observed on baseline screening examinations. On subsequent examinations, women with heterogeneously dense breasts and women aged 50 to 79 years with scattered fibroglandular density benefited from both reduction in recall and increased cancer detection. Women aged 40 to 49 years with scattered fibroglandular density and women aged 50 to 79 years with almost entirely fatty breasts benefited from reduced recall without changes in cancer detection. By contrast, women with extremely dense breasts did not appear to benefit from DBT in terms of recall or cancer detection after the baseline examination.

Our study is unique in its ability to estimate DBT performance by screening round, age group, and breast density categories given our large sample size. Prior studies evaluating performance of DBT by breast density have shown benefits for women with dense breasts, but have largely dichotomized dense (heterogeneously dense and extremely dense) and nondense (almost entirely fat and scattered fibroglandular densities) categories.^1,10,25^ As our results illustrate, there are important differences in performance that may not be appreciated by combining density categories. Our results are consistent with a prospective double-reader study of DM and DBT conducted in Norway^26^ that assessed differences in performance by breast density category assessed using automated volumetric assessment and similarly found no significant change in cancer detection in the highest and lowest density categories. One prior US multi-institutional analysis of DBT performance that examined all density categories reported no change in cancer detection but did observe improved recall rates in women with extremely dense breasts^27^; however, it did not stratify by round of screening.

Our findings suggest that density likely should not be used as a criterion to triage use of DBT for routine screening in settings where DBT is not universally available, as has been reported in physician surveys.^11,12^ Women with nondense breasts experience greater screening benefits with DBT than women with extremely dense breasts, with reductions in screening recall rates and gains in cancer detection. Moreover, the largest absolute improvements of DBT screening were achieved on the baseline screening examination, suggesting that women presenting for their first screening examination are particularly important to prioritize for DBT screening.

Our finding that screening benefits of DBT differ for women with heterogeneously dense breasts vs extremely dense breasts is especially important in the current landscape of density legislation and demand for supplemental screening tests beyond mammography. To date, most state mandates and the proposed federal legislation have uniformly grouped women with heterogeneously dense breasts and those with extremely dense breasts as a single population. Although there is currently no consensus on the optimal approach to screening for women with dense breasts, our findings suggest that women with extremely dense breasts will not benefit from DBT replacing DM for routine breast cancer screening. Thus, these women may benefit more from supplemental screening compared with women with heterogeneously dense breasts. Although studies of supplemental imaging in women with dense breasts have shown improvements in cancer detection with magnetic resonance imaging^28,29,30^ and ultrasonography,^30,31^ they have generally not distinguished outcomes by density category. Density and risk specific performance outcomes from trials of supplemental screening are needed to determine whether future legislative efforts and clinical guidelines could potentially be tailored more narrowly to women with extremely dense breasts, which comprise fewer than 10% of screening-aged women.

Although our study suggests that overall, women benefit from screening DBT, it is important to note the potential drawbacks. Our results suggest that biopsy recommendations increase on baseline examinations and in women with heterogeneously dense breasts. However, many of these biopsies result in an increase in cancers detected by DBT, and the ratio of biopsies performed to cancers detected is similar between the 2 modalities. Screening DBT when performed in combination with DM also results in approximately 2-fold higher doses of radiation exposure when performed in combination with DM.^10,32^ To address this concern, breast imaging facilities are increasingly transitioning to use of synthetic DM reconstructed from the tomosynthesis acquisition,^33^ reducing radiation exposure to doses comparable to conventional DM. Another important drawback is screening costs, which are approximately 40% higher for screening DBT than screening with DM (based on 2019 Centers for Medicare and Medicaid Services fee schedule). Although some of these additional costs are offset by reductions in false-positive examinations, prior work by some members of our team has shown that overall, the costs are likely high relative to the expected improvements in health outcomes.^34^ Currently, these additional costs are frequently incurred by patients because screening with DBT is not always covered by private insurance. By contrast, out-of-pocket expenses for women receiving screening DM are generally prohibited by federal mandate.

### Limitations

It is important to note that owing to our observational study design, conclusions about causality cannot be made. However, we provided performance estimates adjusted for woman-level risk factors and other variables that could confound differences in performance, which is particularly important during a time when both DM and DBT are available and women may be offered DBT screening based on clinical, demographic, or risk factor characteristics. Of note, we chose to exclude facilities with fewer than 100 DM or DBT examinations performed during the study period because of evidence for differences in DM performance between facilities that offer DBT and those that do not;^18^ therefore, our results may not be generalizable to facilities offering DBT exclusively. We also did not distinguish between performance of DBT performed in combination with conventional 2-dimensional mammography vs synthetic 2-dimensional mammography; however, it is unlikely that our results would change with this distinction because studies to date have shown no difference in performance between DBT interpreted with DM vs synthetic mammography.^32,35^

## Conclusions

In conclusion, our results suggest that the largest performance improvements with DBT compared with DM may occur in women undergoing baseline screening mammography. On subsequent screening rounds, women aged 40 to 79 years with heterogeneously dense breasts and women aged 50 to 79 years with scattered fibroglandular density may benefit most from DBT, with both lower recall rates and higher cancer detection rates. Women with extremely dense breasts do not appear to benefit from improvements in recall rates or cancer detection rates with DBT on subsequent screening rounds. Our results provide guidance for women and physicians making decisions regarding use of DBT for routine screening.

## Notes

Supplement. **eTable.** Unadjusted Absolute Rates of Screening Recall, Biopsy Recommendation, and Cancer Detection

## References

[1] Conant EF, Barlow WE, Herschorn SD, ; Population-based Research Optimizing Screening Through Personalized Regimen (PROSPR) Consortium Association of digital breast tomosynthesis vs digital mammography with cancer detection and recall rates by age and breast density. JAMA Oncol. 2019;5(5):635-642. doi:10.1001/jamaoncol.2018.7078 30816931 PMC6512257

[2] Ciatto S, Houssami N, Bernardi D, Integration of 3D digital mammography with tomosynthesis for population breast-cancer screening (STORM): a prospective comparison study. Lancet Oncol. 2013;14(7):583-589. doi:10.1016/S1470-2045(13)70134-7 23623721

[3] Friedewald SM, Rafferty EA, Rose SL, Breast cancer screening using tomosynthesis in combination with digital mammography. JAMA. 2014;311(24):2499-2507. doi:10.1001/jama.2014.6095 25058084

[4] McDonald ES, Oustimov A, Weinstein SP, Synnestvedt MB, Schnall M, Conant EF Effectiveness of digital breast tomosynthesis compared with digital mammography: outcomes analysis from 3 years of breast cancer screening. JAMA Oncol. 2016;2(6):737-743. doi:10.1001/jamaoncol.2015.5536 26893205

[5] Marinovich ML, Hunter KE, Macaskill P, Houssami N Breast cancer screening using tomosynthesis or mammography: a meta-analysis of cancer detection and recall. J Natl Cancer Inst. 2018;110(9):942-949. doi:10.1093/jnci/djy121 30107542

[6] Fujii MH, Herschorn SD, Sowden M, Detection rates for benign and malignant diagnoses on breast cancer screening with digital breast tomosynthesis in a statewide mammography registry study. AJR Am J Roentgenol. 2019;212(3):706-711. doi:10.2214/AJR.18.20255 30673339 PMC6386608

[7] Richman IB, Hoag JR, Xu X, Adoption of digital breast tomosynthesis in clinical practice. JAMA Intern Med. 2019. doi:10.1001/jamainternmed.2019.1058 31233086 PMC6593646

[8] U.S. Food and Drug Administration MQSA national statistics. Accessed August 9, 2019. https://www.fda.gov/Radiation-EmittingProducts/MammographyQualityStandardsActandProgram/FacilityScorecard/ucm113858.htm

[9] Haas BM, Kalra V, Geisel J, Raghu M, Durand M, Philpotts LE Comparison of tomosynthesis plus digital mammography and digital mammography alone for breast cancer screening. Radiology. 2013;269(3):694-700. doi:10.1148/radiol.13130307 23901124

[10] Bernardi D, Macaskill P, Pellegrini M, Breast cancer screening with tomosynthesis (3D mammography) with acquired or synthetic 2D mammography compared with 2D mammography alone (STORM-2): a population-based prospective study. Lancet Oncol. 2016;17(8):1105-1113. doi:10.1016/S1470-2045(16)30101-2 27345635

[11] Gao Y, Babb JS, Toth HK, Moy L, Heller SL Digital breast tomosynthesis practice patterns following 2011 FDA approval: a survey of breast imaging radiologists. Acad Radiol. 2017;24(8):947-953. doi:10.1016/j.acra.2016.12.011 28188043

[12] Hardesty LA, Kreidler SM, Glueck DH Digital breast tomosynthesis utilization in the united states: a survey of physician members of the Society of Breast Imaging. J Am Coll Radiol. 2016;13(11S):R67-R73. doi:10.1016/j.jacr.2016.09.030 27814818

[13] Lee CI, Lehman CD Digital breast tomosynthesis and the challenges of implementing an emerging breast cancer screening technology into clinical practice. J Am Coll Radiol. 2016;13(11S):R61-R66. doi:10.1016/j.jacr.2016.09.029 27814817

[14] Are You Dense Inc State density reporting efforts—because your life matters. Accessed August 9, 2019. https://www.areyoudenseadvocacy.org/dense

[15] US Food and Drug Administration FDA advances landmark policy changes to modernize mammography services and improve their quality. Accessed August 9, 2019. https://www.fda.gov/news-events/press-announcements/fda-advances-landmark-policy-changes-modernize-mammography-services-and-improve-their-quality

[16] Lehman CD, Arao RF, Sprague BL, National performance benchmarks for modern screening digital mammography: update from the Breast Cancer Surveillance Consortium. Radiology. 2017;283(1):49-58. doi:10.1148/radiol.2016161174 27918707 PMC5375631

[17] Kerlikowske K, Molinaro AM, Gauthier ML, Biomarker expression and risk of subsequent tumors after initial ductal carcinoma in situ diagnosis. J Natl Cancer Inst. 2010;102(9):627-637. doi:10.1093/jnci/djq101 20427430 PMC2864293

[18] Miglioretti DL, Abraham L, Lee CI, ; Breast Cancer Surveillance Consortium Digital breast tomosynthesis: radiologist learning curve. Radiology. 2019;291(1):34-42. doi:10.1148/radiol.2019182305 30806595 PMC6438358

[19] Breast Cancer Surveillance Consortium Risk Calculator V2 Published 2018 Accessed August 1, 2019. https://tools.bcsc-scc.org/bc5yearrisk/calculator.htm

[20] D’Orsi CJ, Sickles EA, Mendelson EB, ACR BI-RADS® Atlas, Breast Imaging Reporting and Data System. American College of Radiology; 2013.

[21] D’Orsi CJ, Mendelson EB, Ikeda DM, Breast Imaging Reporting and Data System: ACR BI-RADS–Breast Imaging Atlas. American College of Radiology; 2003.

[22] Liang K-Y, Zeger SL Longitudinal data analysis using generalized linear models. Biometrika. 1986;73(1):13-22. doi:10.1093/biomet/73.1.13

[23] Miglioretti DL, Heagerty PJ Marginal modeling of multilevel binary data with time-varying covariates. Biostatistics. 2004;5(3):381-398. doi:10.1093/biostatistics/kxg042 15208201

[24] le Cessie S Bias formulas for estimating direct and indirect effects when unmeasured confounding is present. Epidemiology. 2016;27(1):125-132. doi:10.1097/EDE.0000000000000407 26426943

[25] McCarthy AM, Kontos D, Synnestvedt M, Screening outcomes following implementation of digital breast tomosynthesis in a general-population screening program. J Natl Cancer Inst. 2014;106(11):dju316. doi:10.1093/jnci/dju316 25313245 PMC4271033

[26] Østerås BH, Martinsen ACT, Gullien R, Skaane P Digital mammography versus breast tomosynthesis: impact of breast density on diagnostic performance in population-based screening. Radiology. 2019;293(1):60-68. doi:10.1148/radiol.2019190425 31407968

[27] Rafferty EA, Durand MA, Conant EF, Breast cancer screening using tomosynthesis and digital mammography in dense and nondense breasts. JAMA. 2016;315(16):1784-1786. doi:10.1001/jama.2016.1708 27115381

[28] Bakker MF, de Lange SV, Pijnappel RM, ; DENSE Trial Study Group Supplemental MRI screening for women with extremely dense breast tissue. N Engl J Med. 2019;381(22):2091-2102. doi:10.1056/NEJMoa1903986 31774954

[29] Comstock CE, Gatsonis C, Newstead GM, Comparison of abbreviated breast MRI vs digital breast tomosynthesis for breast cancer detection among women with dense breasts undergoing screening. JAMA. 2020;323(8):746-756. doi:10.1001/jama.2020.0572 32096852 PMC7276668

[30] Berg WA, Zhang Z, Lehrer D, ; ACRIN 6666 Investigators Detection of breast cancer with addition of annual screening ultrasound or a single screening MRI to mammography in women with elevated breast cancer risk. JAMA. 2012;307(13):1394-1404. doi:10.1001/jama.2012.388 22474203 PMC3891886

[31] Tagliafico AS, Mariscotti G, Valdora F, A prospective comparative trial of adjunct screening with tomosynthesis or ultrasound in women with mammography-negative dense breasts (ASTOUND-2). Eur J Cancer. 2018;104:39-46. doi:10.1016/j.ejca.2018.08.029 30316869

[32] Skaane P, Bandos AI, Eben EB, Two-view digital breast tomosynthesis screening with synthetically reconstructed projection images: comparison with digital breast tomosynthesis with full-field digital mammographic images. Radiology. 2014;271(3):655-663. doi:10.1148/radiol.13131391 24484063

[33] Zuckerman SP, Sprague BL, Weaver DL, Herschorn SD, Conant EF Survey results regarding uptake and impact of synthetic digital mammography with tomosynthesis in the screening setting. J Am Coll Radiol. 2020;17(1, pt A):31-37. doi:10.1016/j.jacr.2019.07.020 31415739 PMC6952532

[34] Lowry KP, Trentham-Dietz A, Schechter CB, Long-term outcomes and cost-effectiveness of breast cancer screening with digital breast tomosynthesis in the United States. J Natl Cancer Inst. 2020;112(6):582-589. doi:10.1093/jnci/djz184 31503283 PMC7301096

[35] Simon K, Dodelzon K, Drotman M, Accuracy of synthetic 2D mammography compared with conventional 2D digital mammography obtained with 3D tomosynthesis. AJR Am J Roentgenol. 2019;212(6):1406-1411. doi:10.2214/AJR.18.20520 30917028

