# Supplementary material for: Screening Performance of Digital Breast Tomosynthesis vs Digital Mammography in Community Practice by Patient Age, Screening Round, and Breast Density
Source: JAMA Netw Open. 2020 Jul 28;3(7):e2011792. doi: 10.1001/jamanetworkopen.2020.11792 (PMC7388021; doi:10.1001/jamanetworkopen.2020.11792)
Supplement: Supplement. — eTable. Unadjusted Absolute Rates of Screening Recall, Biopsy Recommendation, and Cancer Detection [file jamanetwopen-3-e2011792-s001.pdf]

Supplementary Online Content

Lowry KP, Coley RY, Miglioretti DL, et al. Screening performance of digital breast tomosynthesis vs digital mammography in community practice by patient age, screening round, and breast density. *JAMA Netw Open*. 2020;3(7):e2011792. doi:10.1001/jamanetworkopen.2020.11792

eTable. Unadjusted Absolute Rates of Screening Recall, Biopsy Recommendation, and Cancer Detection

This supplementary material has been provided by the authors to give readers additional information about their work.

© 2020 Lowry KP et al. *JAMA Network Open*.

eTable. Unadjusted Absolute Rates of Screening Recall, Biopsy Recommendation, and Cancer Detection

Values are expressed as absolute rates per 1000 screening examinations (95% confidence intervals).

			Recall Rate		Biopsy Recommendation Rate		Total Cancer Detection Rate		Invasive Cancer Detection Rate	
	Age, years	BI-RADS Density	DM	DBT	DM	DBT	DM	DBT	DM	DBT
Baseline	40-49	All	241 (237, 245)	212 (204, 220)	36.1 (34.3, 37.9)	41.8 (37.8, 45.8)	3.1 (2.6, 3.7)	4.6 (3.2, 5.9)	2.2 (1.7, 2.6)	3.0 (1.9, 4.1)
	50-59		241 (235, 248)	202 (190, 215)	40.8 (37.8, 43.8)	56.4 (49.1, 63.7)	6.0 (4.9, 7.2)	8.1 (5.3, 11.0)	4.2 (3.3, 5.2)	6.6 (4.0, 9.1)
	60-79		221 (214, 227)	171 (158, 184)	40.8 (37.6, 43.9)	48.4 (41.2, 55.6)	10.8 (9.1, 12.4)	15.4 (11.3, 19.5)	8.8 (7.3, 10.3)	13.4 (9.5, 17.2)
Subsequent	40-49	Almost entirely fat	58 (55, 62)	46 (38, 53)	7.5 (6.1, 8.8)	8.6 (5.3, 11.9)	0.5 (0.1, 0.8)	0.8 (0.0, 2.0)	0.4 (0.1, 0.7)	0.4 (0.0, 1.2)
		Scattered fibroglandular density	104 (102, 106)	77 (73, 80)	13.6 (12.9, 14.4)	11.2 (9.7, 12.6)	2.3 (2.0, 2.6)	2.0 (1.3, 2.6)	1.6 (1.4, 1.9)	1.6 (1.0, 2.2)
		Heterogeneously dense	133 (131, 135)	113 (110, 116)	17.8 (17.1, 18.5)	19.7 (18.1, 21.2)	2.5 (2.2, 2.8)	3.1 (2.4, 3.7)	1.6 (1.4, 1.8)	2.2 (1.7, 2.8)
		Extremely dense	113 (110, 116)	117 (110, 123)	19.0 (17.6, 20.3)	19.1 (16.4, 21.8)	2.9 (2.4, 3.5)	2.8 (1.7, 3.9)	1.7 (1.3, 2.1)	1.5 (0.7, 2.2)
	50-59	Almost entirely fat	47 (45, 49)	37 (33, 41)	9.1 (8.2, 10.1)	7.4 (5.5, 9.3)	1.9 (1.5, 2.4)	1.3 (0.4, 2.2)	1.4 (1.0, 1.8)	0.8 (0.1, 1.5)
		Scattered fibroglandular density	83 (81, 84)	65 (63, 68)	13.0 (12.4, 13.5)	12.8 (11.7, 13.8)	3.3 (3.0, 3.6)	3.5 (2.8, 4.1)	2.4 (2.2, 2.7)	2.7 (2.2, 3.3)
		Heterogeneously dense	103 (102, 105)	88 (86, 91)	16.9 (16.2, 17.6)	18.0 (16.7, 19.3)	3.8 (3.5, 4.1)	5.3 (4.5, 6.1)	2.7 (2.4, 2.9)	4.1 (3.5, 4.8)
		Extremely dense	92 (89, 95)	89 (82, 95)	19.3 (17.7, 20.8)	16.8 (13.9, 19.7)	4.1 (3.4, 4.9)	4.7 (3.0, 6.3)	2.6 (2.0, 3.2)	2.9 (1.6, 4.1)
	60-79	Almost entirely fat	51 (50, 53)	37 (34, 40)	12.6 (11.7, 13.4)	9.6 (8.0, 11.2)	4.3 (3.8, 4.8)	3.5 (2.4, 4.6)	3.5 (3.0, 3.9)	3.1 (2.0, 4.1)
		Scattered fibroglandular density	75 (74, 76)	58 (56, 60)	14.4 (13.9, 14.8)	13.0 (12.1, 13.9)	5.5 (5.2, 5.8)	6.5 (5.8, 7.2)	4.3 (4.0, 4.6)	5.0 (4.4, 5.6)
		Heterogeneously dense	88 (87, 90)	73 (70, 75)	16.4 (15.7, 17.1)	17.5 (16.2, 18.8)	6.1 (5.7, 6.5)	7.6 (6.6, 8.5)	4.6 (4.3, 5.0)	6.1 (5.3, 7.0)
		Extremely dense	65 (61, 69)	59 (52, 66)	12.6 (10.9, 14.3)	12.2 (9.1, 15.4)	4.6 (3.5, 5.6)	4.8 (2.6, 7.0)	3.0 (2.1, 3.8)	4.0 (2.1, 6.0)

Abbreviations: DM=Digital mammography; DBT=Digital breast tomosynthesis; BI-RAD=Breast Imaging Reporting and Data System

© 2020 Lowry KP et al. *JAMA Network Open*.
